# TP53 Co-Mutations in Advanced EGFR-Mutated Non–Small Cell Lung Cancer: Prognosis and Therapeutic Strategy for Cancer Therapy

**DOI:** 10.3389/fonc.2022.860563

**Published:** 2022-04-04

**Authors:** Surui Liu, Jin Yu, Hui Zhang, Jie Liu

**Affiliations:** ^1^Department of Oncology, Jinan Central Hospital, Jinan, China; ^2^Department of Oncology, Shandong First Medical University & Shandong Academy of Medical Sciences, Jinan, China; ^3^Department of Thyroid Surgery, Shandong Provincial Hospital Affiliated to Shandong First Medical University, Jinan, China

**Keywords:** non-small cell lung cancer (NSCLC), EGFR, TP53, comutation, primary resistance, treatment

## Abstract

Lung cancer is the leading cause of cancer-related deaths worldwide. As the most prevalent molecular mutation subtypes in non-small cell lung cancer (NSCLC), EGFR-TKIs are currently a standard first-line therapy for targeting the mutated EGFR in advanced NSCLC patients. However, 20-30% of this subset of patients shows primary resistance to EGFR-TKIs. Patients with co-mutations of EGFR and several other genes have a poor response to EGFR-TKIs, whereas the prognostic and predictive significance of EGFR/TP53 co-mutation in NSCLC patients remains controversial. Meanwhile, little is known about how to choose an optimal therapeutic strategy for this subset of patients. Presently, no drugs targeting TP53 mutations are available on the market, and some p53 protein activators are in the early stage of clinical trials. A combination of EGFR-TKIs with antiangiogenic agents or chemotherapy or other agents might be a more appropriate strategy to tackle the problem. In this review, we describe the prognostic and predictive value of EGFR/TP53 co-mutation in NSCLC patients, investigate the mechanisms of this co-mutation affecting the response to EGFR-TKIs, and further explore optimal regimens effectively to prolong the survival time of the NSCLC patients harboring this co-mutation.

## Introduction

Lung cancer is the second most commonly diagnosed malignancy worldwide and the leading cause of cancer-related death in 2020 (1). Non-small cell lung cancer(NSCLC) is the most common type of lung cancer, accounting for about 85-90% of lung cancer cases (2). The majority of patients present with advanced stages at initial diagnosis. Available treatments include chemotherapy, immunotherapy, and target therapy.

Epidermal growth factor receptor (EGFR) mutations are the most prevalent driver mutations in patients with NSCLC (3). An exon 19 short deletion (E746–A750) and an exon 21 point mutation, L858R, termed classical mutations, are the most common mutations accounting for about 85-90% (4). Atypical mutations include G719X, S768I, L861Q, and other point mutations (5), accounting for about 6-12% of EGFR mutations (6, 7). EGFR mutations occur most frequently in lung adenocarcinomas, women, never-smokers, and in Asian populations (6). Initially, first and second-generation EGFR-tyrosine kinase inhibitors (EGFR-TKIs) were approved as the standard for first-line treatment for patients with classical EGFR mutations (exon 19 deletions and L858R) whose median progression-free survival (mPFS) ranged from 9.2 to 12 months (8–11), and median overall survival (mOS) (22.1-28.2 months) (11–13). Subsequently, third-generation TKIs were confirmed as the first-line treatment with mPFS 18.9-19.3 months (14, 15), and mOS 33.1-38.6 months (15, 16).

However, 20-30% of patients with this subset of patients showed primary resistance to EGFR-TKIs (17). The understanding of the mechanisms underlying this primary resistance would be a prerequisite to overcoming the resistance and improving the therapeutic outcome of those patients. In recent years, accumulating research has focused on the molecular mechanisms of the acquired resistance to EGFR-TKIs and exploring alternative therapeutic strategies. Relatively few studies have been conducted on the mechanism of the primary EGFR-TKIs resistance. Uncommon EGFR mutations, such as *de novo* T790M mutation or exon 20 insertion mutation, could result in primary resistance to EGFR-TKIs (18). In addition, alternative pathway activation including BRAF mutation, HER2 mutation, KRAS mutation, and phosphatidylinositol3-kinase (PIK3CA) mutation, also caused primary resistance (19–22). With the development of next-generation sequencing (NGS), more and more co-mutations in advanced EGFR mutated-NSCLC patients were found, and these co-mutations might be one of the mechanisms of primary drug resistance, among which TP53 mutations were the most frequent co-mutations, accounting for about 17.3-72.5% (19, 23). Therefore, we will review the prognostic and predictive value of EGFR/TP53 co-mutation in NSCLC patients, investigate the mechanisms of this co-mutation affecting the response to EGFR-TKIs, and further explore optimal regimens effectively to prolong the survival time the NSCLC patients harboring this co-mutation.

## Prognostic and Predictive Value of EGFR/TP53 Co-mutation in Advanced NSCLC Patients

TP53 gene, located on chromosome 17 short arm (17p13), is a tumor suppressor gene encoding tumor protein p53, which consists of 393 amino acids with four domains: an activation domain at the N-terminus (TAD), a central DNA-binding domain (DBD), a tetramerization domain (TD) in its C-terminal domain (CTD) and an extreme CTD regulatory domain. Protein p53 is involved in many biological processes, including DNA repair, metabolism, cell cycle arrest, apoptosis, and aging (24). TP53 mutations were associated with poor prognosis in a wide variety of cancers (25–31). In NSCLC, TP53 mutations were detected in approximately 40% of lung adenocarcinoma and 51% of squamous cell carcinoma (32, 33). Previous reports indicated that 17.3-72.5% of advanced EGFR-mutant lung cancers harbor TP53 mutations (19, 23).

Canale et al. found that disease control rate (DCR) was 70% in TP53-mutated patients compared to 88% in TP53-wild type (TP53-wt) patients [relative risk, RR, of disease progression: 3.17 (95% CI 1.21-8.48), P=0.019] in 123 EGFR-mutated NSCLC patients receiving TKIs as first-line therapy (17). Subsequently, shorter mPFS (6.5 months VS 14.0 months, P=0.025) and mOS (28.0 months VS 52.0 months, P=0.023) were discovered in TP53 mutant patients treated with first-line TKIs compared with TP53 wild-type patients (34). In another study, TP53 co-mutation was discovered as a predictor for TKI efficacy and survival in EGFR+ NSCLC irrespective of other currently available parameters and it might be an important factor for risk stratification of newly diagnosed metastatic EGFR+ NSCLC (35). Several other researchers also obtained similar results (36, 37) (**Table 1**). In addition, Ying Cheng et al. integrated the genomic data and clinical outcomes in 179 patients with advanced EGFR-mutated NSCLC and found that EGFR-mutant patients harboring concomitant TP53 mutation were significantly associated with a poorer clinical prognosis (OS: 21 vs. 40 months, P = 0.05) after treated with 1st generation EGFR-TKI. In contrast, the presence of TP53 mutation did not affect the PFS or OS of patients treated with 2nd generation EGFR-TKI (38).

**Table 1:** TP53 co-mutation as a poor prognostic factor in EGFR-mutated NSCLC patients.

Study	TP53-mut vs. TP53-wt				EGFR-TKI Predictor
	mOS (months)	mPFS (months)	DCR	ORR	
Canale 2017 (17)	N/A	N/A	70% vs. 88% (P=0.019)	N/A	YES
Hou 2019 (34)	28 vs. 52 (P=0.023)	6.5 vs. 14 (P=0.025)	76.7% vs. 89.3% (P=0.160)	25 vs. 28% (P=0.374)	YES
Christopoulos 2020 (35)	P<0.05	P<0.001	N/A	N/A	YES
Yu 2018 (37)	NR vs. 47 (P=0.036)	N/A	N/A	N/A	YES
Cheng 2020 (38)	21 vs. 40 (P=0.05)	11.2 vs. 13.1 (P=0.2)	N/A	N/A	YES (1^st^ generation TKI) NO (2^nd^ generation TKI)

N/A, No Available.

There were also several other studies failing to identify an adverse prognostic value of TP53 mutation (**Table 2**). Catherine et al. found that concomitant TP53 mutation status was not associated with relapse-free survival (RFS) or OS in patients with EGFR-mutant NSCLC at an early stage who underwent primary surgical resection and received adjuvant chemotherapy, suggesting that co-mutations were not a strong prognostic marker in early-stage patients without receiving EGFR-TKI therapy (39). Meanwhile, it was also found that objective response rate (ORR) was not significantly different (TP53-mut 54%, wt 66%, P=0.42) and there was a non-significant trend towards shorter mPFS on EGFR with TP53 mutation (HR 1.74, CI 0.98–3.10, P=0.06) in advanced NSCLC patients who received EGFR-TKI treatment (39). The work by Ying Jin et al. and Anna et al. supported this, showing that no significant difference in PFS was observed for TP53 co-mutation in advanced EGFR-mutated lung adenocarcinomas (19, 23). Therefore, the utility of EGFR/TP53 co-mutation as a prognostic and predictive biomarker for advanced EGFR-mutated NSCLC patients remains controversial.

**Table 2:** TP53 co-mutation is not associated with prognosis in EGFR-mutated NSCLC patients.

Study	TP53-mut vs. TP53-wt			EGFR-TKI Predictor
	mOS (months)	mPFS (months)	ORR	
Rachiglio 2019 (23)	18.9 vs. 23 (P = 0.19)	12.3 vs. 9.9 (P = 0.29)	N/A	N/A
Labbé 2017 (39)	70.2 vs.71.2 (P = 0.39)	10.0 vs. 16.8 (P = 0.06)	54% vs. 66% (P = 0.42)	YES

N/A, No Available.

A growing number of studies were conducted to determine whether the prognostic and predictive effect of TP53 mutations varied by type of gene mutation (**Table 3**). On basis of mutations subtypes, TP53 mutations showed a remarkable preference for missense mutations over nonsense and frameshift mutations that are commonly dominant in other tumor suppressor genes such as RB1, adenomatous polyposis coli (APC), and PTEN (44). One study showed that NSCLC patients with TP53 missense mutations had significantly shorter PFS treated with first-line EGFR-TKI therapy (HR 1.91, CI 1.01–3.60, P=0.04) (39). In another published study, TP53 non-missense mutations reduced responsiveness to first-generation TKIs and worsen the prognosis of EGFR-mutant advanced NSCLC patients (mPFS: 6.3 vs 14 months, P=0.041; HR 2.01, 95% CI: 1.00–4.05, P=0.046; mOS: 21.2 vs 52.5 months, P=0.001; HR 5.53, 95% CI: 1.79–16.95 P=0.001) (34). According to the functional effects on the p53 protein, TP53 mutations were divided into disruptive mutations and non-disruptive mutations (45). Disruptive mutations likely led to complete or almost complete loss of p53 protein activity, while non-disruptive mutations could retain some functional properties of wt-p53 (45). Molina-Vila’s study showed that non-disruptive mutations were predictive of shorter survival in the EGFR-mutated patients both in the training and in the validation cohorts (40). However, they found no significant association in erlotinib-treated patients carrying non-disruptive mutations (40). Multiple subsequent studies have demonstrated the prognostic roles of TP53 non-disruptive mutations. Meanwhile, it was found that these non-disruptive mutations could predict the response to first-line TKIs treatment in EGFR-mutated NSCLC patients (17, 25, 34). However, an analysis based on the cBioPortal database collected 1441 pieces of data from 1441 metastatic NSCLC patients and showed no prognostic value of disruptive or non-disruptive mutation of TP53 (41). Increasing research focused on the mutations based on the location of the mutation. The most common mutation site of TP53 is exon 4-8, accounting for about 44.8% (41), and exon 5-8 encodes DBD and recognizes consistent sequences in promoters of multiple genes involved in widely disparate biological effects (46, 47). Yang JJ et al. analyzed data obtained from a phase III randomized clinical trial (CTONG 0901) and found that mPFS in patients with mutations in exon 4 or 7 of TP53, other TP53 mutations, and wild-type TP53 were 9.4, 11.0, and 14.5 months (P=0.009), respectively. mOS were 15.8, 20.0, and 26.1 months (P=0.004), respectively. Mutations in exon 4 or 7 of TP53 served as independent prognostic factors for PFS (P=0.001) and OS (P=0.004) in advanced EGFR-mutant patients (42). Hou’s study consistently concluded that the mutation of exon 7 in exons 5-8 of TP53 greatly shortened the prognosis of patients compared with the wt-TP53 control group (mPFS: 5.0 vs 14.0 months, P=0.002, HR 3.98, 95% CI: 1.53-10.31, P=0.002, mOS: 14.0 vs 52.5 months, P=0.008, HR 5.29, 95% CI: 1.32-20.83, P=0.009) (34). However, another study showed that patients with exon 4, exon 6, mutation of the unknown site, and multiple mutations of TP53 demonstrated worse prognosis than with exon 5, exon 7, exon 8, and exon 9 mutation in EGFR exon 19/21 mutated patients (41). In Canale’s study, compared with patients with mutations in other TP53 exons and wt-TP53, patients with TP53 exon 8 mutation had shorter PFS (5.8 vs 12.4 vs 14.4 months), confirming the negative effect of exon 8 mutation on PFS (HR3.16,95%1.59-6.28, P=0.001) (43). In addition, it has also been shown that the T790M positive patients with TP53 R237C mutation failed to benefit from the subsequent osimertinib treatment and TP53 rs55863639 polymorphism was associated with the worse prognosis in TP53/EGFR co-mutation samples (48, 49). Thus, different classifications of TP53 mutations might result in different prognostic and predictive outcomes. How to identify the classification warrants further investigation in future studies.

**Table 3:** OS and PFS in different types of TP53 mutation in EGFR-mutated NSCLC patients.

Study	Comparison	mOS (months)	mPFS (months)
Hou 2019 (34)	Missense mut vs. wt	28 vs. 52.5 (P=0.150)	8.4 vs.14 (P=0.068)
Labbé 2017 (39)	Missense mut vs. wt	P= 0.46	P=0.04
Hou 2019 (34)	Non-missense mut vs. wt	21.2 vs. 52.5 (P=0.001)	6.3 vs. 14 (P=0.041)
Yu 2021 (25)	Non-disruptive mut vs. wt	21.73 vs. 31.6 (P<0.001)	7.57 vs. 13 (P=0.01)
Hou 2019 (34)	Non-disruptive mut vs. wt	35 vs. 52.5 (P=0.008)	6.3 vs. 14 (P=0.028)
Molina-Vila 2014 (40)	Non-disruptive mut vs. wt	17.8 vs. 28.4 (P=0.04)	11 vs. 15 (P=0.14)
Jiao 2018 (41)	Non-disruptive mut vs. disruptive mut	21 vs. 18 (P=0.69)	N/A
Yang 2020 (42)	Exon 4/7 vs. other mut vs. wt	15.8 vs. 20.0 vs. 26.1 (P=0.004)	9.4 vs. 11.0 vs. 14.5 (P=0.008)
Hou 2019 (34)	Exon 7 vs. wt	14 vs. 52.5 (P=0.009)	5 vs. 14 (P=0.002)
Jiao 2018 (41)	Exon5/7-9 vs. exon4/6/multiple exon mut/unknown exon mut vs. wt	21 vs. 13 vs. 27 (P<0.001)	N/A
Canale2020 (43)	Exon 8 vs. other mut vs. wt	18.53 vs. 34.8 vs. 27.3 (P=0.440)	5.8 vs. 14.4 vs. 12.4 (P=0.002)

N/A, No Available.

## Mechanisms of the EGFR/TP53 Co-mutation Affecting the Response to EGFR-TKIs in Advanced EGFR-Mutated NSCLC Patients

The negative prognostic effect of TP53 mutations might be attributed to their tumor-suppressive function loss, genomic instability function gain, and abilities of cancer cell transcriptome and phenotype regulation (40, 50, 51). However, the underlying mechanism of TP53 concurrent mutations as primary resistance to EGFR-TKIs in patients with advanced NSCLC remains poorly understood. A previous study investigated the role of p53 in growth-inhibitory and apoptotic effects of gefitinib in the human NSCLC cell lines NCI-H1299 and A549, which have no EGFR mutations and found that p53 enhanced gefitinib-induced growth inhibition and apoptosis by regulation of Fas (factor associated suicide) in NSCLC (52). However, whether the same mechanisms were shared in EGFR mutations was unknown. Another study showed that p53-knocked cells represented a significant reduction in sensitivity to EGFR inhibitors, compared to their parental cells. Conversely, restoration of functional p53 in EGFR inhibitor-resistant cells was sufficient to resensitize them to EGFR inhibitors or radiation *in vitro* and *in vivo (* 53). The above findings demonstrated that tumor-suppressive functions loss of p53 determined EGFR inhibitors sensitivity.

Although these functions are traditionally thought of as the major functions of the p53 protein for tumor suppression, recent research has suggested that mutant p53 proteins showed oncogenic gain-of-function (GOF) properties, such as promoting tumor progression and acquiring drug resistance (40, 54, 55). Patricia et al. showed that mutant p53 expression can promote invasion, loss of directionality of migration, and metastatic behavior by constitutive activation of EGFR/integrin signaling (56). Molina-Vila’s confirmed that at least 11 non-disruptive mutations have induced GOF activity in cell models, and promoted tumor progression through the down-regulation of apoptosis and cell block genes, and the up-regulation of mitosis, angiogenesis, or drug-resistant tyrosine kinase receptor (Axl) genes (40, 57).

A bioinformatics study showed that in dual mutation samples, differential expression genes (DEGs) were strikingly enriched in regulating the metabolism of important amino acids, cell division, cell cycle regulation, cell adhesion, and extracellular matrix composition which were mainly enriched in signaling pathways such as PI3K-Akt, cytokine–cytokine receptor interaction, focal adhesions, and extracellular matrix receptor interaction, and PPI network suggested that GPC3, CCL28, GPR37, and NPY genes were up-regulated (58). Another study analyzed the data of 491 patients from The Cancer Genome Atlas (TCGA) and demonstrated that co-mutation of EGFRL858R/TP53 increased the expression of COMP and ITGB8, which are involved in an extracellular matrix organization and cell surface receptor signaling pathways, thus contributing to poor prognosis in lung adenocarcinoma. Validation was performed using three GEO profiles and similar results were obtained. CCK-8 and cell colony formation assays indicated that comutant EGFRL858R/TP53MUT promoted cell proliferation ability compared to the cowild A549 NC and TP53MUT H1299 NC. Additionally, the results revealed that EGFRL858R/TP53MUT resulted in increasing migration abilities compared with A549 and H1299 cells in the NC group (49). Although the exact mechanism of EGFR/TP53 co-mutation on prognosis is not specified, the aforementioned studies indicate that they may depend, at least partially, on the cellular functions and pathways.

In addition, Michael Offin et al. found that patients with EGFR/RB1/TP53-mutant lung cancers had a shorter time to discontinuation than EGFR/TP53- and EGFR-mutant-only cancers(9.5 versus 12.3 versus 36.6 months, respectively, P=2×10^-9^). It was demonstrated that TP53 inactivation and RB1 loss might be early events of small cell transformation which would generate resistance to TKIs (59).

## Clinical Implications in Advanced EGFR-Mutated NSCLC Patients With TP53 Co-mutation

Recent studies suggested that the current routine testing of EGFR for selecting NSCLC patients treatable with first-line targeted therapy was not enough to predict the response to the TKIs (32). As mentioned above, patients with TP53 co-mutation in advanced EGFR-mutated NSCLC showed poor prognosis and insensitivity to EGFR-TKIs. However, little was known about how to choose the best treatment modality in the aforementioned populations. At present, no drugs targeting TP53 mutations are available on the market, and some p53 protein activators, such as ARP-246, are in the early stage of clinical trials (60). A combination of EGFR-TKI therapy with chemotherapy or antiangiogenic agents or other agents might be a more appropriate strategy to tackle the problem. Thus, this paper will explore the combination treatment options in patients with TP53 mutations to provide therapeutic strategies for clinicians.

### Combination of EGFR-TKIs and Chemotherapy

Before 2011, platinum-based chemotherapy was the cornerstone of treatment of advanced NSCLC. As the driver oncogenes were identified, therapeutic strategies for NSCLC, especially for those with driver mutations have been revolutionized in recent years. EGFR TKIs have become a standard of first-line treatment for patients with EGFR-mutated NSCLC. Recently, a randomized phase III study (NEJ009) showed that compared with gefitinib alone, gefitinib combined with carboplatin plus pemetrexed improved PFS in patients with untreated advanced NSCLC with EGFR mutations (20.9 vs 11.2 months, P < 0.001) (9). The results from a phase II study (JMIT) supported this observation as well, showing that pemetrexed + gefitinib improved PFS (15.8 vs 10.9 months, P=0.028) compared with gefitinib alone in East Asian patients with advanced NSCLC and activating EGFR mutations (61).

Could patients with TP53 co-mutation benefit from combined treatment with EGFR-TKIs and chemotherapy? Here we first reviewed the effects of the sensitivity of tumor cells to chemotherapy by the status of p53. *In vitro* experiments showed that NCI-H1299 (p53-null) cells were insensitive to cisplatin (CDDP) (62, 63). Similar results were observed in other studies. Cisplatin (CDDP) significantly induced apoptosis in A549 (p53-wt) cells, but not in H1299 cells, and p53-deficient tumor cells show chemoresistance to drugs, suggesting that a functional p53 might affect the chemosensitivity of NSCLC (64, 65). One study was analyzed for the p53 genotype in patients with advanced NSCLC who had received neoadjuvant chemotherapy and found that the presence of a mutant p53 genotype was highly indicative of resistance to induction chemotherapy (P<0.002) (66). Another study showed that in advanced NSCLC the response rate of the p53 positive group was 26% versus 57% of the p53 negative group (P=0.004), and in multivariate analyses, positive p53 was identified as an independent predictive factor for resistance to cisplatin-based chemotherapy(P=0.006) (67). However, some studies were inconsistent with the above observation, which demonstrated that TP53 mutations were not significant predictive of response to cisplatin-based chemotherapy (68–70). To date, no studies have been published for EGFR-TKIs plus chemotherapy in advanced EGFR/TP53 co-mutation NSCLC. In ClinicalTrials.gov, one registered phase III clinical study comparing osimertinib monotherapy to combination therapy with osimertinib, carboplatin, and pemetrexed for untreated patients with advanced non-squamous NSCLC with concurrent EGFR and TP53 mutations is currently undergone. We can look forward to the final results of this study in the coming years.

### Combination of EGFR-TKIs and Antiangiogenic Drugs

Since the Eastern Cooperative Oncology Group (ECOG) 4599 study showed a significant survival benefit with the use of bevacizumab in combination with carboplatin and paclitaxel in comparison with CP chemotherapy alone in patients with previously untreated advanced, metastatic or recurrent NSCLC (71), antiangiogenic drugs were increasingly being used in clinical practice (72).

Antiangiogenic drugs mainly targeted vascular endothelial growth factor (VEGF)/vascular endothelial growth factor receptor (VEGFR) signal pathway in tumor angiogenesis. A previous study showed that TP53 mutation was significantly associated with higher VEGF expression level (P=0.006) (73), and mutant p53 binding to the VEGFR2 promoter transcription start site, regulated angiogenesis by promoting VEGFR2 up-regulation (74).

So far, several studies have suggested TP53 mutation might be predictive of clinical sensitivity to antiangiogenic therapies in different tumor types. One prospective study analyzed outcomes based on VEGF/VEGFR inhibitor treatment and the presence of TP53 mutations in various tumor types. The results showed that treatment with VEGF/VEGFR inhibitors was independently associated with improvement in all outcome parameters [rate of SD≥6 months/PR/CR, length of TTF and OS (all P≤ 0.01)] for the patients harboring TP53-mutant cancers, but the improvement was not seen in any of these parameters for the group of patients with TP53 wild-type neoplasms (75). Another study retrospectively reviewed 19 cases of patients with advanced sarcoma treated with VEGFR inhibition and discovered that the PFS of patients with TP53 mutations was significantly greater than TP53 wild-type tumors with the median PFS of 208 versus 136 days, respectively [P=0.036, hazards ratio 0.38 (95% confidence interval 0.09-0.83)] (76). Similar findings were reported in non-small cell lung cancer. Said with his colleagues retrospectively analyzed the response to standard systemic therapy of 145 patients with documented tumor p53 mutational status (mutant-type [mtp53] vs. wild-type [wtp53]), and the results showed that PFS was significantly longer with bevacizumab-containing regimens as compared to non-bevacizumab containing regimen in patients with mtp53 (median 11.0 [95% CI 5.9-16.0], n=22 vs. 4.0 months [95% CI: 3.6-5.7], n=35, P<0.0001), but not those with wtp53 (median 5.0 [95% CI: 2.0-7.6] vs. 6.0 [95% CI 4.0-7.5] months, P=0.318) (77). Anlotinib, a novel oral multi-target antiangiogenic TKI directed against VEGFR-1/2/3, fibroblast growth factor receptor (FGFR), was approved as a third-line treatment for advanced NSCLC in China (78). Fang discussed three NSCLC patients with TP53 mutation treated with anlotinib as a second or third- line regimen, all three patients achieved PR and PFS of 8 months, 6.5 months, and 5 months respectively (79).

However, the above-mentioned studies were conducted without knowing EGFR mutation status. For patients with EGFR mutation, multiple studies had demonstrated that antiangiogenic agents plus EGFR-TKIs significantly improved PFS compared with EGFR-TKIs alone (80–84). Were similar results obtained in EGFR-mutated patients with TP53 concurrent mutations? Results from RELAY showed that a combination of the antiangiogenic agent ramucirumab with EGFR-TKI targeted therapy provided significant and similar clinical benefit for both EGFR ex19del and ex21L858R NSCLC, and its subgroup analyses showed that in patients with co-mutations of EGFR and TP53, ramuzumab plus erlotinib showed better PFS treatment advantage than erlotinib alone (P<0.001) (85). A single-center retrospective analysis by Cheng Y et al. recruited 179 patients with advanced EGFR-mutated NSCLC, and the results showed that bevacizumab combined with EGFR-TKI regimen could significantly improve PFS (14 vs 9.7months, P=0.034) in patients with TP53 co-mutation compared with EGFR-TKI single drug (38). At present, for this clinical condition, combination therapies of EGFR-TKIs and antiangiogenic agents might provide a better benefit for patients with TP53 co-mutation.

### Combination of EGFR-TKIs and Immunotherapy

Immune checkpoint inhibitors, including monoclonal antibodies against programmed death-1 (PD-1) and programmed death ligand-1 (PD-L1), either as monotherapy or combination therapy, have made a breakthrough in clinical treatment for patients with advanced NSCLC (86). However, subgroup analyses from several clinical trials demonstrated that no PFS and/or OS benefit was achieved from anti-PD-1/PD-L1 therapy in the patients harboring EGFR mutations (87–89). Based on the above trials, immunotherapy is not recommended as the preferred treatment of patients with NSCLC harboring EGFR mutation by the NCCN guidelines at present. Meanwhile, safety concerns caused by the combination of EGFR-TKIs and anti-PD-1/PD-L1 therapy resulted in the termination of some clinical trials (90, 91). Thus, the combination of EGFR-TKI and immunotherapy could not achieve the expected therapeutic value.

Are all advanced NSCLC patients with EGFR mutation not suitable for immunotherapy? In the phase II ATLANTIC study, the investigators assessed the effect of durvalumab (anti-PD-L1) treatment as third-line or later treatment in three cohorts of patients with NSCLC defined by EGFR/ALK status and tumor expression of PD-L1. The proportion of patients with EGFR-/ALK- NSCLC achieving a response was higher than that with EGFR+/ALK+NSCLC. The ORR among the patients with EGFR+ NSCLC with ≥ 25% of tumor cells expressing PD-L1 remained encouraging (12.2%) relative to that (4%) among patients with < 25% PD-L1 expression (92). Therefore, how to choose the proper patients suitable for durvalumab in the EGFR+NSCLC patients warrants further investigation.

Patients with EGFR/TP53 co-mutation might gain little benefit from EGFR-TKIs treatment, could they benefit from ICIs? So far no definitive biomarker for immunotherapy has been established. Currently, PD-L1 expression status in tumor tissue is now considered a predictive biomarker for selecting patients who could benefit the most (93). A retrospective study examined tumor PD-L1 expression and eleven gene mutations in 247 surgically resected primary and 26 advanced NSCLC patients and revealed that PD-L1 expression was significantly associated with TP53 mutation (P=0.014) using multivariate logistic regression (94). Similar results were obtained by Yoon JC et al. (95). Moreover, Hao Sun et al. demonstrated that the TP53-missense-mutation group showed increased PD-L1 (CD274) level and enriched IFN-γ signatures compared with the TP53-wild-type subgroup, but no differences were noted in patients with nonsense-mutant vs wild-type p53 (96). Furthermore, a real-world study of a large Chinese cohort suggested that mutations in TP53 were significantly associated with high PD-L1 expression (both P < 0.001). High PD-L1 expression was significantly associated with EGFR co-mutation with tumor suppressor genes such as TP53, while EGFR mutation alone was not associated with high PD-L1 expression, and these results might explain why advanced NSCLC patients with EGFR mutation alone showed poor response to immunotherapy and patients with EGFR/TP53 co-mutation were likely to benefit from anti-PD-1/PD-L1 treatment (97). The tumor mutation burden (TMB) is another important biomarker used to predict the response to cancer immunotherapy in NSCLC patients (98). One study investigated the relationship between TMB and the imaging, histologic, and genetic features in NSCLC and found that tumors with high TMB were more prevalent in those with a TP53 mutation (P<0.0001) (99).

Emerging evidence from a few recent clinical studies suggested that advanced TP53-mutated NSCLC patients could benefit from immunotherapy. Sandra et al. revealed that in advanced NSCLC patients treated with nivolumab, with or without CTLA-4 blocker ipilimumab, or pembrolizumab, the median OS in the TP53-mutated group was 18.1 months (95% CI 6.6-not reached) vs 8.1 months (95% CI 2.2–14.5, hazard ratio [HR] = 0.48; 95% CI 0.25-0.95, P=0.04) in the TP53-wild-type group and the mPFS was significantly longer in TP53-mutated patients (4.5 months, 95% CI 2.8–18.1 versus 1.4, 95% CI 1.1–3.5; P=0.03). In multivariate analysis, TP53 mutations were independently associated with longer OS (HR = 0.35, 95% CI 0.16-0.77, P=0.009), whereas TP53 mutation status failed to significantly influence PFS (P=0.32) (100). Another study showed that TP53-mutated lung adenocarcinoma treated with ICIs showed significantly prolonged PFS (P=0.017, HR=0.69 [95%CI: 0.50-0.94]) (101). Dong et al. retrospectively showed in 30 NSCLC patients treated with pembrolizumab that median PFS was significantly longer in the TP53-mutated group than in the TP53-wild-type group (14.5 versus 3.5 months, P=0.042) (102). Jun Lu et al. also indicated that the potential predictors of immunotherapy were significantly different, especially between patients with TP53 (+) and TP53 (−) (103). Alternatively, it has been shown that TP53 missense but not nonsense mutants were associated with better clinical benefits taking antiPD-1/L1. However, all such TP53 subgroups responded well to nivolumab (antiPD-L1) plus ipilimumab (antiCTLA-4) therapy (96). An increasing number of recent studies have demonstrated that TP53 and other gene co-mutations might serve as a predictive biomarker for ICI responses in NSCLC. Dong ZY et al. observed that the TP53/KRAS co-mutated subgroup manifested exclusive increased expression of PD-L1 and the highest proportion of PD-L1+/CD8A+, increased mutation burden, and specifically enriched in the transversion-high (TH) cohort. And it was also found that TP53 or KRAS mutation patients, especially those with co-occurring TP53/KRAS mutations, showed remarkable clinical benefit to PD-1 inhibitors (102). Another subsequent research suggested that the presence of KRAS/STK11 co-mutation and KRAS/STK11/TP53 co-mutation affected OS only in patients treated with ICIs (HR=10.936, 95% CI: 2.337-51.164, P=0.002; HR=17.609, 95% CI: 3.777-82.089, P<0.001, respectively) (104). One multiple-cohort study included patients with NSCLC from the Gene plus Institute, the Cancer Genome Atlas (TCGA), and the Memorial Sloan Kettering Cancer Center (MSKCC) databases and from the POPLAR and OAK randomized controlled trials and found that TP53 and ataxia-telangiectasia mutated (ATM) co-mutation was associated with a significantly higher TMB compared with the sole mutation and with no mutation. Among patients treated with ICIs in the POPLAR and OAK cohort, TP53 and ATM co-mutation was associated with better PFS and OS than a single mutation and no mutation groups (mPFS: TP53 and ATM co-mutation, 10.4 months; TP53 mutation, 1.6 months; ATM mutation, 3.5 months; no mutation, 2.8 months; P=0.01; mOS: TP53 and ATM co-mutation, 22.1 months; TP53 mutation, 8.3 months; ATM mutation, 15.8 months; no mutation, 15.3 months; P=0.002) (105). In addition, TP53/KMT2C co-mutation was also considered a potential predictive factor in guiding anti-PD-1/PD-L1 immunotherapy (106). Based on these previous results, immunotherapy could be a valuable option for advanced NSCLC patients with EGFR/TP53 co-mutation.

However, how could we improve the clinical outcome of ICIs, monotherapy, or in combination with other agents, such as another ICI, conventional chemotherapy, and antiangiogenic drugs? The IMpower150 trial could provide some implications for us. In this phase III study participants with chemotherapy-naive metastatic non-small-cell lung cancer were randomly assigned (1:1:1) to receive atezolizumab plus bevacizumab plus carboplatin plus paclitaxel (ABCP), atezolizumab plus carboplatin plus paclitaxel, or the standard-of-care bevacizumab plus carboplatin plus paclitaxel (BCP) every three weeks. Efficacy was assessed in a key subgroup with EGFR mutations previously treated with one or more tyrosine kinase inhibitors within the intention-to-treat population. It was found that improved OS with ABCP versus BCP was observed in patients with EGFR mutations in the intention-to-treat population (19.8 months vs 14.9 months; HR 0.76, 95% CI: 0.63-0.93) (107). Despite the limited sample size of EGFR-mutated patients, this result could indicate that patients with EGFR‐TKI resistance might benefit from the combination of ICIs, antiangiogenic drugs, and conventional chemotherapy. In summary, further investigations will be warranted within current molecular stratification for appropriate therapeutic regimens.

## Conclusion

So far, most studies suggested that TP53 mutations were an important marker of poor prognosis and predictor in advanced EGFR-mutated NSCLC. However, the classification of TP53 mutations was very complicated, and which mutation contributed to prognosis and prediction, had not been consistently concluded. The answers to these inconsistencies will be further investigated in future studies. Although the exact mechanism of EGFR/TP53 co-mutation on prognosis is not specified, current studies indicate that they may depend, at least partially, on the cellular functions and pathways and small cell transformation generating resistance to TKIs. At present, to this clinical condition, combinations with immunotherapy or combination therapies of EGFR-TKIs and antiangiogenic agents may be valuable options for the advanced NSCLC patients with EGFR/TP53 co-mutation. However, all of these studies were retrospective, and most with small patient numbers. Thus, further verification using a large sample size and stratifying patients based on TP53 mutation status in studies, especially prospective studies are needed in the future.

## Author Contributions

All authors contributed to the article and approved the submitted version.

## Funding

This study was funded by the National Natural Science Foundation of China (81501989), the Natural Science Foundation of Shandong Province (ZR2020MH210, ZR2020QH179), and the Jinan Medical and Health Science and Technology Development Project (grant no. 201907118).

## Conflict of Interest

The authors declare that the research was conducted in the absence of any commercial or financial relationships that could be construed as a potential conflict of interest.

## Publisher’s Note

All claims expressed in this article are solely those of the authors and do not necessarily represent those of their affiliated organizations, or those of the publisher, the editors and the reviewers. Any product that may be evaluated in this article, or claim that may be made by its manufacturer, is not guaranteed or endorsed by the publisher.
